# Supplementary material for: The effects of genital myiasis on the diversity of the vaginal microbiota in female Bactrian camels
Source: BMC Vet Res. 2022 Mar 5;18:87. doi: 10.1186/s12917-022-03189-5 (PMC8897907; doi:10.1186/s12917-022-03189-5)
Supplement: Supplementary file 5 — Additional file 5. [file 12917_2022_3189_MOESM5_ESM.zip › MPL201709200_16s_yy/Treat1/C02_plsda/plsda.pdf]

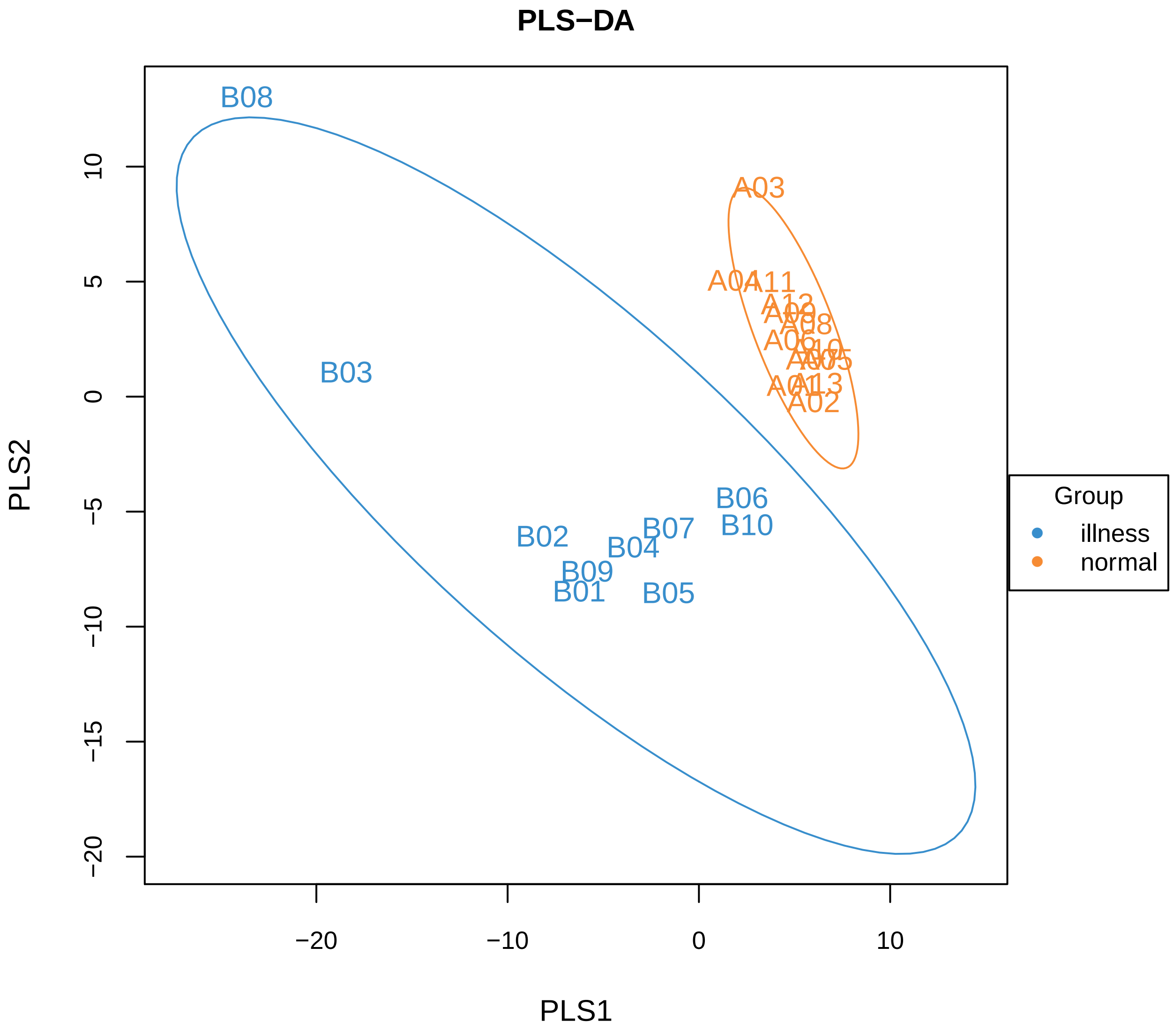
PLS−DA
PLS1
PLS2
−20
−10
0
10
5
−5
−15
B08
B03
B06
B10
B07
B02
B04
B09
B01
B05
A03
A04
A11
A02
Group
illness
normal
